# Development of omega‐3 rich eggs through dietary flaxseed and bio‐evaluation in metabolic syndrome

**DOI:** 10.1002/fsn3.1522

**Published:** 2020-04-30

**Authors:** Hira Shakoor, Muhammad Issa Khan, Amna Sahar, Muhammad Kashif Iqbal Khan, Furukh Faiz, Hafiz Basheer Ahmad

**Affiliations:** ^1^ National Institute of Food Science and Technology University of Agriculture Faisalabad Pakistan Faisalabad; ^2^ Department of Food Engineering University of Agriculture Faisalabad Faisalabad Pakistan; ^3^ Department of Agriculture and Food Technology Karakoram International University Gilgit Pakistan; ^4^ Ayub Agriculture Research Institute Faisalabad Pakistan

**Keywords:** high‐density lipoprotein, low‐density lipoprotein, metabolic syndrome, omega‐3 fatty acid, very low‐density lipoprotein

## Abstract

An egg is a nutrient‐dense food that contains protein, fats, vitamins, and minerals. It is proven that the consumption of eggs influences serum lipid concentration. Therefore, a study was conducted to investigate the effect of normal and omega‐3 eggs on serum lipids profiles. Lipids were extracted from egg yolks and analyzed for fatty acids content. The present research is a crossover study design in which 20 participants were recruited randomly, and all subjects received three treatments: no eggs, omega‐3 eggs, and normal eggs. However, fasting blood was drawn at baseline and the end of each diet period and analyzed for serum lipids, blood glucose, and insulin level. Omega‐3 egg treatment showed reduction in the serum total cholesterol by 16.57 mg/dl (*p* < .001), triglyceride by 17.48 mg/dl, and increase in HDL cholesterol concentration by 0.48 mg/dl (*p* < .001) as compared to no‐egg. A significant (*p* < .05) reduction in blood pressure by 8.34/8.67 mm/Hg and insulin level was observed due to omega‐3 egg consumption which indicates that omega‐3 fatty acids improve insulin sensitivity. On the other hand, regular egg intake elevates serum total cholesterol and triglycerides concentration but decreases blood pressure. It was concluded that omega‐3‐enriched egg consumption had a positive effect on the serum lipid profile and blood pressure of patients with metabolic syndrome as compared to normal eggs.

## Graphical abstract

Consumption of eggs either omega‐3‐enriched or conventional has a positive potential toward cholesterol, HDL cholesterol, and blood pressure

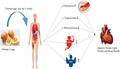

## 1 INTRODUCTION

A metabolic syndrome is a group of metabolic risk factors such as obesity, glucose intolerance, dyslipidemia, and insulin intolerance, that affects approximately 20%–25% of the adult population in the world (Bhowmik et al., 2015). Diet plays a vital role in protecting the body from these metabolic anomalies, while functional food provides additional health benefits besides basic nutrients (Khan, Khan, et al., 2017). Different bioactive components of the diet are considered to regulate metabolic syndrome, including phospholipids, omega‐3 fatty acids, arginine, folate, some minerals, and so forth (Fuller, Sainsbury, Caterson, & Markovic, 2015). Among these healthy diet components, omega‐3 fatty acids, such as alpha‐linolenic acid (ALA), eicosapentaenoic acid (EPA), and docosahexaenoic acid (DHA), are proven to have a potential mechanistic approach against multiple risk factors of metabolic syndrome including hyperlipidemia, hypertension, high blood glucose, hepatic steatosis, and endothelial dysfunction (Dasarathy et al., 2015). An egg can contribute 72 kcal and has some healthy elements, including protein, bioactive lipids (phospholipids), some fat‐soluble vitamins (A, D, E, and K), vitamin B complex, and minerals (Andersen, 2015; Larsson, Akesson, & Wolk, 2015). Enrichment of eggs with omega‐3 contents is inexpensive way to make it readily available for everyone. Addition of flaxseed in hen's diet increases the amount of omega‐3 fatty acids, *that is,* docosahexaenoic acid (DHA) and alpha‐linolenic acid (ALA). These omega‐3‐enriched eggs contain up to five times more omega‐3 fatty acid constituents compared to normal eggs (Egg Nutrition Council, 2014). In order to reduce the cardiometabolic risk, it is essential to have a diet that is rich in omega‐3 fatty acids (Yashodhara et al., 2009). Evidence showed that omega‐3 fatty acids are involved in reverse cholesterol transport, improve vascular compliance, and decrease arterial stiffness. Hence, it reduces the risk of atherosclerosis and insulin resistance (Blesso, Andersen, Barona, Volek, & Fernandez, 2013; Papanikolaou, Brooks, Reider, & Fulgoni, 2014; Tousoulis et al., 2014). The omega‐3 fatty acids are present in eggs which help in reducing the level of cholesterol, triglycerides, and the particle size of low‐density lipoprotein (LDL) by converting them to the less atherogenic elements (Lewis, Schalch, Scheideler, 2000; Lewis, Seburg, & Flanagan, 2000). A 13% triacylglycerol reduction was found in a group that consumed omega‐3 eggs, while 18% triacylglycerol reduction was observed in group consuming walnuts (Burns‐Whitmore, Haddad, Sabate, & Rajaram, 2014). Moreover, egg phospholipids help in the reduction of lipid absorption, C‐reactive protein (CRP), and the alpha factor of tumor necrosis in plasma (Blesso, 2015; Compos et al., 2016).

Unhealthy dietary habits and sedentary lifestyle increase the risk of metabolic syndrome, diabetes, hypertension, and cardiovascular disease (Fuller et al., 2015). To deal with the increasing risk of diseases, the present study was conducted to evaluate the effect of omega‐3 eggs in patients with metabolic syndrome.

## 2 MATERIALS AND METHODS

### 2.1 Production of omega‐3‐enriched eggs

The study was undertaken at National Institute of Food Science and Technology, University of Agriculture Faisalabad. The omega‐3‐enriched eggs were produced under the project of “Development of conditioned (omega‐3‐rich) meat and eggs through modification in feed ingredients.” Omega‐3‐enriched eggs were produced by adding 20% flaxseed to the hens' diet. Omega‐3 in the hen diet boost omega‐3 in egg yolk up to 220 mg which is equal to 100 g lean fish (Hargis & Van Elswyk, 1993).

### 2.2 Fatty acids profile of eggs

Lipids from egg yolks were extracted by following the method described by Folch, Lees, and Sloane‐Stanley (1957). Esters of fatty acids were prepared and analyzed using gas chromatography according to the (Ce if‐96) method given in AOCS (American Oil & Chemist Society, 1998).

### 2.3 Subjects

This study was a randomized crossover (3 × 3 Latin square), free‐living intervention experiment in which subjects were blinded to egg treatments. Twenty‐one subjects aged 30–40 years old, diagnosed with metabolic syndrome, were included in this study. The selection criteria for patients were according to the International Diabetes Federation (IDF) consensus which states obesity: waist circumference 94 cm or more for men and 80 cm or more for women, waist‐to‐hip ratio (WHR) 0.90 cm or above for males and 0.85 or above for females, and body mass index (BMI) 30 or above. Hypertension: systolic BP measurement of 130 mmHg or more and diastolic BP measurement of 85 mmHg or above. High blood glucose level: fasting blood glucose of 100 mg/dl (5.6 mmol/L) or more, but the patient is not suffering from type 2 diabetes mellitus. Dyslipidemia: blood triglycerides level of 150 mg/dl (1.7 mmol/L) or more, or may use cholesterol‐lowering medicine such as statins. Dyslipidemia: low level of high‐density lipoprotein (HDL) cholesterol which is less than 40 mg/dl (1.04 mmol/L) for men and less than 50 mg/dl (1.3 mmol/L) for women, or the use of cholesterol‐lowering medicine like statins (Bhowmik et al., 2015). Patients with egg allergy, insulin‐dependent diabetes mellitus, heart diseases, stroke, acute or chronic insufficiency, cancer, liver failure, and endocrine disorders were excluded from the study.

### 2.4 Treatment plan

Three treatments were used in this study: no eggs, two boiled omega‐3 eggs per day, and two boiled normal eggs per day at breakfast. Subjects were blinded to the egg treatment and randomly assigned to the treatment in a manner consistent with the Latin square protocol. The study design was a crossover (3 × 3 Latin square) such that each subject received each of the three treatments. Before the study, the baseline value of serum lipid profile, blood pressure, glucose, and insulin level was recorded, and every individual served as his or her own control. Each treatment was conducted for 5 weeks, with a five‐week washout period between treatments, in order to minimize the possible carryover effects. Exercise and medication were maintained during the study period.

### 2.5 Anthropometrics and blood pressure assessment

Subjects' weight (kg) and height (m) were measured to calculate their body mass index (BMI) (kg/m^2^), as described by Keys, Fidanza, Karvonen, Kimura, and Taylor (1972). Waist and hip circumference of the subjects was estimated according to the criteria described by Skorkowska‐Telichowska (2016). Blood pressure was monitored after 15 min of rest by a standardizing mercury manometer placed on the midupper arm of the subject following the method of Oh, Ryue, Hsieh, and Bell (1991).

### 2.6 Plasma lipids profile

Fasting blood samples of the subjects, collected by syringe at the beginning of the experiment as a baseline value and at the end of each treatment period, were analyzed for serum lipids. Serum lipid profile, including serum total cholesterol and triglycerides, were analyzed by the enzymatic method of Allian, Poon, Chan, Richmond, and Fu (1974) and Nagele et al. (1984). High‐density lipoprotein (HDL) cholesterol was measured directly after the precipitation of other lipoproteins by dextran sulfate (Warnick, Nguyen, & Albers, 1985). Low‐density lipoprotein (LDL) and very low‐density lipoprotein (VLDL) were calculated. Plasma LDL and VLDL cholesterol concentrations were measured using the Friedewald, Levy, and Fredrickson (1972) equation as follows:

VLDL = TRIG/5 and LDL = TC − (VLDL + HDL) as mentioned in Nijke, Faridi, Dutta, Gonzalez‐Simon, and Katz (2010).

The normal range of total cholesterol is <200 mg/dl, triglyceride is 50–150 mg/dl, HDL is 35–60 mg/dl, and LDL is <150 mg/dl.

### 2.7 Glucose and insulin level

The blood sugar level of the subjects was determined using ACCU‐CHEK (Dacus, Schulz, Averill, & Sibai, 1989), whereas the insulin level was measured following the method of Ballesteros et al. (2015).

### 2.8 Statistical analysis

The data were subjected to statistical analysis using SPSS 23 and Minitab 17. The crossover (3 × 3 Latin square) design was used to determine the significance level. When the trend was significant, the difference among treatments was analyzed by the Tukey HSD test and *p* < .05, which was adjusted for multiple comparisons.

## 3 RESULTS

Among the 21 eligible participants at start of the study, one patient was dropped out due to time constraints and lack of motivation to continue the study. About 20 individuals including 11 men and 9 women completed all three treatments. Clinical trials were conducted on patients with metabolic syndrome to determine the impact of eggs on the serum lipid profile. However, the results regarding each parameter are summarized as follows.

### 3.1 Fatty acids composition of eggs

Gas chromatography was used to analyze the FAME of eggs and identify different types of fatty acids including C18:3, C18:2, C20:5, C22:6, and C22:5. The 20% flaxseed in the hens' diet showed an increase in the level of polyunsaturated omega‐3 fatty acids C18:3 by 7.39%, C20:5 by 1.53%, C22:6 by 6.20%, C22:5 by 0.33%, and a slight increase in omega‐6 fatty acid by 9.61% compared to the control eggs. As shown in Table 1, in modified eggs (omega‐3 egg), the percentage of omega‐3 and omega‐6 fatty acid was highly significant as compared to normal eggs.

**TABLE 1:** PUFA composition (%) of normal and omega‐3‐rich egg yolks

Fatty acid	T1 (%)	T2 (%)	*p* value
C18:3 (*n* − 3)	1.16 ± 0.04^b^	7.39 ± 0.08^a^	.000**
C18:2 – Cis (*n* − 6)	7.71 ± 0.19^b^	9.61 ± 0.07^a^	.004*
C20:5 (*n* − 3)	0.76 ± 0.07^b^	1.53 ± 0.03^a^	.003*
C22:6 (*n* − 3)	0.33 ± 0.03^b^	6.20 ± 0.03^a^	.000**
C22:5 (*n* − 3)	0.06 ± 0.002^b^	0.33 ± 0.006^a^	.000**

Note

All the values are mean ± *SE*; *n* = 20, means within the same row having different superscripts are significantly different (*p* < .05).

T1, normal eggs, T2, omega‐3 fatty acid.

### 3.2 Anthropometric measurements

Participants included in the study were overweight because their BMI and WHR were higher than normal ranging from 28 to 30 and 0.92 to 0.95, respectively. Consumption of normal and omega‐3 eggs does not have significant effect on BMI and WHR of patients with metabolic syndrome (Table 2.)

**TABLE 2:** Anthropometric measurements at end of each treatment

	No‐egg	Omega−3 egg	Normal egg	*p* value
WHR	0.920 ± 0.016	0.938 ± 0.016	0.920 ± 0.016	.161
BMI	30.210 ± 1.044	29.933 ± 1.044	28.904 ± 1.044	.274

Note

All the values are mean ± *SE*; *n* = 20, means within the same row having different superscripts are significantly different by Tukey's HSD (*p* < .05).

Abbreviations: BMI, body mass index; WHR, Waist‐to‐hip ratio.

### 3.3 Serum lipid profile

The comparison of normal egg and no‐egg treatments with omega‐3 egg treatment showed a significant decrease in total cholesterol from 46.14 to 16.57 mg/dl, respectively (*p < *.001). Taking two omega‐3 eggs in breakfast reduce total cholesterol and triglycerides by 12.5% and 10.5%, respectively as shown in Table 3, whereas normal egg consumption increases total cholesterol to 227.523 mg/dl. Moreover, omega‐3 eggs resulted in increase of HDL level by 9.3% compared to normal eggs (Mutungi et al., 2008). On the other hand, no change was seen in treatment of normal eggs. A similar trend was observed in serum LDL and VLDL cholesterol concentration, in which a slight reduction was observed due to omega‐3‐enriched eggs compared to no‐egg and normal egg consumption. However, the result is statistically insignificant.

**TABLE 3:** Serum lipid level of individuals at end of each treatment

	No‐egg	Omega−3 egg	Normal egg	*p* value
Cholesterol (mg/dl)	197.952 ± 8.527^b^	181.381 ± 8.527^a^	227.523 ± 8.527^c^	.001**
Triglycerides (mg/dl)	169.952 ± 7.489	152.476 ± 7.489	187.333 ± 7.489	.082
HDL (mg/dl)	46.619 ± 2.790^a^	47.095 ± 2.790^a^	42.904 ± 2.790^b^	.010*
LDL (mg/dl)	139.333 ± 16.500	98 ± 16.500	124.366 ± 16.500	.072
VLDL (mg/dl)	35.900 ± 5.242	31.166 ± 5.242	41.600 ± 5.242	.180

Note

All the values are mean ± *SE*; *n* = 20, means within the same row having different superscripts are significantly different by Tukey's HSD (*p* < .05).

### 3.4 Blood pressure, glucose, and insulin level

The lowest value of blood pressure (82/122 mm/Hg) was observed in omega‐3 egg treatment, and the highest value of (90/130) mm/Hg was seen in the no‐egg treatment. A significant (*p* < .001) reduction of blood pressure by 8.34/8.67 mm/Hg due to omega‐3 egg consumption. Interestingly, improvement in blood pressure was also seen in normal egg treatment (128/85 mm/Hg) compared to the no‐egg, as shown in Table 4.

**TABLE 4:** Blood pressure, glucose and insulin level at the end of each treatment

	No‐egg	Omega−3 egg	Normal egg	*p* value
Diastolic (mm/Hg)	90 ± 1.708^c^	81.667 ± 1.708^a^	85 ± 1.708^b^	.001**
Systolic (mm/Hg)	130 ± 1.849^b^	121.667 ± 1.849^a^	128.333 ± 1.849^b^	.001**
Glucose (mg/dl)	117.833 ± 3.237	111.833 ± 3.237	114 ± 3.237	.845
Insulin level (u IU/ml)	20.083 ± 2.311^b^	18.700 ± 2.311^a^	24.667 ± 2.311^c^	.058*

Note

All the values are mean ± *SE*; *n* = 20, means within the same row having different superscripts are significantly different by Tukey's HSD (*p* < .05)

Omega‐3 eggs improve glucose and insulin levels by 4% and 14%, respectively, indicating improvement in insulin sensitivity. The insulin level of patients with metabolic syndrome exhibited a significant (*p* < .05) difference among all three groups.

## 4 DISCUSSION

The nutraceutical value and health benefits of eggs can be enhanced by adapting appropriate feeding strategies in poultry as well as by developing designer eggs like omega‐3‐enriched eggs (Laudadio et al., 2015). Feed modification of the hens elevated the omega‐3 contents in the egg yolk. However, a previous study investigated that feed modification of hen with flaxseed increases ALA to 200 mg per egg and raises the amount of DHA content up to 90 mg per egg (Lemahieu et al., 2015). Our findings showed that the addition of 20% flaxseed in hens' diet increases linolenic acid, EPA, and DHA in egg yolks significantly as compared to normal eggs, which is similar to the findings of Al‐Nasser et al. (2011).

Anthropometric measurements are an essential parameter for the identification of individuals with metabolic syndrome. Addition of two boiled eggs (omega‐3 and normal) in diet did not significantly affect BMI and WHR but previous studies showed a change in anthropometric measurement due to egg consumption (Blesso, Andersen, Barona, Volek, et al., 2013; Skorkowska‐Telichowska et al., 2016). Nevertheless, polyunsaturated fatty acid (PUFA) serves as good fats for human health; therefore, increasing PUFA contents in the egg yolk helps to decrease the bad cholesterol (Fraeye et al., 2012). Our finding reported a reduction in serum cholesterol and triglyceride concentration by 12.5% and 10.5%, respectively, due to omega‐3 egg consumption. Omega‐3 fatty acids and phospholipids present in omega‐3 egg yolk help to alter the mobilization and metabolism of cholesterol and hence decrease cholesterol level (Jiang, Noh, & Koo, 2001; Khan, Hegde, Wagh, Khan, & Jamadar, 2017; Noh & Koo, 2003). However, normal eggs are rich in cholesterol and can contribute 32% of total dietary cholesterol (Hu et al., 1999). That is why consumption of normal eggs can increase total serum cholesterol as compared to no‐egg (Chakrabarty et al., 2002; Rouhani, Rashidi‐Pourfard, Salehi‐Abargouei, Karimi, & Haghighatdoost, 2017; Severins, Mensink, & Plat, 2015). Weggeman et al. meta‐analysis of clinical trials observed that 100 mg dietary cholesterol from eggs increases plasma total cholesterol by 0.057 mmol/L (Weggemans, Zock, & Katan, 2001).

National Lipid Association (NLA), American College of Cardiology/American Heart Association (ACC/AHA) recommends omega‐3 fatty acid as the first‐line option for patients with hypertriglyceridemia to decrease triglyceride level (Jacobson et al., 2014; Stone et al., 2014). Omega‐3 egg treatment reduces triglyceride level by 17 mg/dl in patients with metabolic syndrome compared to the no‐egg. However, normal eggs enhanced serum triglyceride level. Likewise, evidence proved that linolenic acid‐enriched eggs decreased triglycerides concentration, thereby reducing the risk of cardiovascular diseases (Ferrier et al., 1992; Lewis, Schalch, Scheideler, 2000; Lewis, Seburg, et al., 2000), and these findings are in line with our results. Further, omega‐3 egg consumption in hypertriglyceridemic participants for 6 weeks lowers triglycerides and increases HDL cholesterol as compared to regular eggs (Maki et al., 2003). Evidence showed that omega‐3‐enriched eggs enhanced HDL cholesterol concentration (Andersen et al., 2013; Farrell, 1998; Lee, Lewis, Scheideler, & Carr, 2003; Techakriengkrai, Klangjareonchai, Pakpeankitwattana, Sritara, & Roongpisuthipong, 2012). Undoubtedly, omega‐3 PUFA increases systemic lipoprotein lipase activity that has hypotriacylglycerolaemic effect. For instance, omega‐3 fatty acids decrease the availability of circulating triglycerides that eventually reduce the hepatic synthesis of LDL and VLDL cholesterol (Buckley, Shewring, Turner, Yaqoob, & Minihane, 2004). In the present study, normal egg consumption slightly decreases LDL cholesterol as compared to no‐egg, but the notable reduction was observed with omega‐3 egg consumption. VLDL did not show a significant trend with egg consumption, and its value was highest among normal egg treatments. The results of our studies are similar to the finding of Techakriengkrai et al. (2012). Similarly, it demonstrated that daily egg consumption showed no significant change in LDL cholesterol of the participants.

In the present study, consumption of two omega‐3 eggs reduced diastolic and systolic by 13.6% and 6%, respectively. It is proven that omega‐3 fatty acids help to improve vascular health and arterial stiffness which are associated with metabolic syndrome (Chong, Lockyer, Saunders, & Lovegrove, 2010). Previous studies found that omega‐3 eggs decrease blood pressure (Djousse, Gaziano, Buring, & Lee, 2009; Oh et al., 1991; Stupin et al., 2018). Another condition of metabolic syndrome is high blood glucose level. The present study reported that omega‐3 eggs and normal eggs do not significantly affect the glucose level. Similar results were found in the previous clinical trials that showed no improvement in blood glucose level with daily egg consumption in patients with type 2 diabetes (Djousse et al., 2009; Njike et al., 2010). Contrarily, Pourafshar et al. findings showed a reduction in glucose level (Pourafshar et al., 2016). However, in our study, patients with metabolic syndrome who consumed omega‐3 eggs had improved insulin levels by 14%, and this is statistically significant compared to other treatments. In another research, Samimi, Jamilian, Asemi, and Esmaillzadeh (1988) examined the effect of omega‐3 egg consumption on blood glucose and insulin level in a female with gestational diabetes over a 6‐week period which proved the positive effects of omega‐3 on insulin resistance but had no effect on glucose level which is in line with our findings. Furthermore, evidence showed increase in insulin sensitivity by consumption of an egg‐based breakfast, which proved the beneficial effect of eggs (Clayton et al., 2015).

The present study is a free‐living intervention study in which subjects were not forced to control their diet. However, the previous studies were controlled feeding trial, which mainly follow the dietary guidelines of National Cholesterol Education Program (NCEP) diet (that suggests 30% of total calories come from fat, 10% from saturated, and less than 300 mg/day of dietary cholesterol) and carbohydrate‐restricted diet (Blesso, Andersen, Barona, Volk, et al., 2013; Lewis, Schalch, Scheideler, 2000; Lewis, Seburg, et al., 2000). Further, the effect of omega‐3 eggs along with normal eggs on all parameters of the metabolic syndrome was observed and a positive association was identified between egg consumption and human health. In contrast to prior studies that have focused on the lipid parameters, we also measured the impact of egg treatments on blood pressure, glucose level, and insulin level and found that omega‐3 eggs were good for hypertensive and prediabetic patients. Although the limitation of this study was that a small number of patients were recruited, which may be insufficient to evaluate the change in lipid profile, blood pressure, and glucose level, hence, further studies are needed to confirm these findings using a larger sample size in a real‐life setting.

## 5 CONCLUSIONS

In light of sustained uncertainties and absence of observational evidence about the influence of omega‐3 egg consumption on serum lipid profile, the present study was conducted to identify the potential benefits of eggs. Omega‐3 eggs are nutritious food which occupies an essential place in the food chain, and it also acts as a functional food. Advancements in technology and modern living style increase the consumption of saturated fats day by day and sedentary lifestyle. Replacement of saturated fats with healthy monounsaturated and polyunsaturated fatty acids can help to reduce the risk of diseases. Production of omega‐3‐enriched eggs is a good strategy to include omega‐3 fatty acids in the diet at low cost. Consumption of omega‐3‐enriched eggs has a positive potential toward the reduction of cholesterol, triglycerides, LDL cholesterol, blood pressure, and increase HDL cholesterol concentration. On the other hand, normal eggs elevate serum cholesterol, triglyceride, and VLDL level due to high content of cholesterol in the yolk. Therefore, omega‐3‐enriched eggs are proved to be beneficial for heart health and for the reduction of many life‐threatening diseases.

## CONFLICT OF INTEREST

The authors declare that they do not have any conflict of interest.

## AUTHORS’ CONTRIBUTIONS

HS, MIK, and AS conceived and designed the study. HS conducted the human study, analyzed the fatty acid contents in eggs and biochemical test of patients, and prepared the results and figures. MIK and AS supervised the practical work. HS wrote and finalized the manuscript. AS reviewed the manuscript for possible improvement. MKIK and HBA checked English grammar, statistical analysis, text, organization, and typesetting of the paper. All authors read and approved the final manuscript.

## ETHICAL APPROVAL

Experiments were conducted in accordance with the declaration of Helsinki principles for human subjects. The protocol was approved by the Institutional Bioethical Committee, National Institute of Food Science and Technology, and University of Agriculture Faisalabad, Pakistan. All subjects were briefed about study, and written consent was obtained from all study participants.

## References

[fsn31522-bib-0001] Allain, C. , Poon, L. S. , Chan, C. S. , Richmond, W. F. P. C. , & Fu, P. C. (1974). Enzymatic determination of total serum cholesterol. Clinical Chemistry, 20, 470–475. 4818200

[fsn31522-bib-0002] Al‐Nasser, A. Y. , Al‐Saffar, A. E. , Abdullah, F. K. , Al‐Bahouh, M. E. , Ragheb, G. , & Mashaly, M. M. (2011). Effect of adding flaxseed in the diet of laying hens on both production of omega‐3 enriched eggs and on production performance. International Journal of Poultry Science, 10, 825–831.

[fsn31522-bib-0003] American Oil and Chemist Society (AOCS) (1998). AOCS Official methods Ce if‐96. Determination of cis‐ and trans‐Fatty Acids in Hydrogenated and Refined Oils and Fats by Capillary GLC”, 5th Ed. Edited by D. Firestone. Champaign, IL. USA..

[fsn31522-bib-0004] Andersen, C. J. (2015). Bioactive egg components and inflammation. Nutrients, 7, 7889–7913. 26389951 10.3390/nu7095372 PMC4586567

[fsn31522-bib-0005] Andersen, C. J. , Blesso, C. N. , Lee, J. , Barona, J. , Shah, D. , Thomas, M. J. , & Fernandez, M. L. (2013). Egg consumption modulates HDL lipid composition and increases the cholesterol‐accepting capacity of serum in metabolic syndrome. Lipids, 48, 557–567. 23494579 10.1007/s11745-013-3780-8 PMC3869568

[fsn31522-bib-0006] Ballesteros, M. , Valenzuela, F. , Robles, A. , Artalejo, E. , Aguilar, D. , Andersen, C. , … Fernandez, M. (2015). One egg per day improves inflammation when compared to an oatmeal‐based breakfast without increasing other cardiometabolic risk factors in diabetic patients. Nutrients, 7, 3449–3463. 25970149 10.3390/nu7053449 PMC4446761

[fsn31522-bib-0007] Bhowmik, B. , Afsana, F. , Siddiquee, T. , Munir, S. B. , Sheikh, F. , Wright, E. , … Hussain, A. (2015). Comparison of the prevalence of metabolic syndrome and its association with diabetes and cardiovascular disease in the rural population of Bangladesh using the modified National Cholesterol Education Program Expert Panel Adult Treatment Panel III and International Diabetes Federation definitions. Journal of Diabetes Investigation, 6, 280–288. 25969712 10.1111/jdi.12268 PMC4420559

[fsn31522-bib-0008] Blesso, C. N. (2015). Egg phospholipids and cardiovascular health. Nutrients, 7(4), 2731–2747. 25871489 10.3390/nu7042731 PMC4425170

[fsn31522-bib-0009] Blesso, C. N. , Andersen, C. J. , Barona, J. , Volek, J. S. , & Fernandez, M. L. (2013). Whole egg consumption improves lipoprotein profiles and insulin sensitivity to a greater extent than yolk‐free egg substitute in individuals with metabolic syndrome. Metabolism, 62(3), 400–410. 23021013 10.1016/j.metabol.2012.08.014

[fsn31522-bib-0010] Blesso, C. N. , Andersen, C. J. , Barona, J. , Volk, B. , Volek, J. S. , & Fernandez, M. L. (2013). Effects of carbohydrate restriction and dietary cholesterol provided by eggs on clinical risk factors in metabolic syndrome. Journal of Clinical Lipidology, 7, 463–471. 24079288 10.1016/j.jacl.2013.03.008

[fsn31522-bib-0011] Buckley, R. , Shewring, B. , Turner, R. , Yaqoob, P. , & Minihane, A. M. (2004). Circulating triacylglycerol and apoE levels in response to EPA and docosahexaenoic acid supplementation in adult human subjects. British Journal of Nutrition, 92, 477–483. 15469651 10.1079/bjn20041235

[fsn31522-bib-0012] Burns‐Whitmore, B. , Haddad, E. , Sabate, J. , & Rajaram, S. (2014). Effects of supplementing n‐3 fatty acid enriched eggs and walnuts on cardiovascular disease risk markers in healthy free‐living lacto‐ovo‐vegetarians: A randomized, crossover, free‐living intervention study. Nutrition Journal, 13, 29–36. 24673793 10.1186/1475-2891-13-29 PMC4001106

[fsn31522-bib-0013] Campos, A. M. , Ricardo, F. , Alves, E. , Reis, A. , Couto, D. , Domingues, P. , & Domingues, M. R. M. (2016). Lipidomic investigation of eggs' yolk: Changes in lipid profile of eggs from different conditions. Food Research International, 89, 177–185. 28460903 10.1016/j.foodres.2016.07.006

[fsn31522-bib-0014] Chakrabarty, G. , Bijlani, R. L. , Mahapatra, S. C. , Mehta, N. , Lakshmy, R. , Vashisht, S. , & Manchanda, S. C. (2002). The effect of ingestion of egg on serum lipid profile in healthy young free‐living subjects. Indian Journal of Physiology and Pharmacology, 46, 492–498. 12683227

[fsn31522-bib-0015] Chong, M. F. F. , Lockyer, S. , Saunders, C. J. , & Lovegrove, J. A. (2010). Long chain n−3 PUFA‐rich meal reduced postprandial measures of arterial stiffness. Clinical Nutrition, 29, 678–681. 20199827 10.1016/j.clnu.2010.02.001

[fsn31522-bib-0016] Clayton, Z. S. , Scholar, K. R. , Shelechi, M. , Hernandez, L. M. , Barber, A. M. , Petrisko, Y. J. , … Kern, M. (2015). Influence of resistance training combined with daily consumption of an egg‐based or bagel‐based breakfast on risk factors for chronic diseases in healthy untrained Individuals. Journal of the American College of Nutrition, 34, 113–119. 25785868 10.1080/07315724.2014.946622

[fsn31522-bib-0017] Dacus, J. , Schulz, K. , Averill, A. , & Sibai, B. (1989). Comparison of capillary Accu‐Chek blood glucose values to laboratory values. American Journal of Perinatology, 6, 334–336. 2730738 10.1055/s-2007-999608

[fsn31522-bib-0018] Dasarathy, S. , Dasarathy, J. , Khiyami, A. , Yerian, L. , Hawkins, C. , Sargent, R. , & McCullough, A. J. (2015). Double blind randomized placebo controlled clinical trial of omega 3 fatty acids for the treatment of diabetic patients with nonalcoholic steatohepatitis. Journal of Clinical Gastroenterology, 49, 137–144. 24583757 10.1097/MCG.0000000000000099 PMC4147029

[fsn31522-bib-0019] Djousse, L. , Gaziano, J. M. , Buring, J. E. , & Lee, I. M. (2009). Egg consumption and risk of type 2 diabetes in men and women. Diabetes Care, 32, 295–300. 19017774 10.2337/dc08-1271 PMC2628696

[fsn31522-bib-0020] Egg Nutrition Council . (2014). Position Statement for Healthcare Professional. Eggs and Omega‐3s. Available from: https://www.enc.org.au/position‐statements/eggs‐and‐omega‐3s [accessed 23 Jan 2018].

[fsn31522-bib-0021] Farrell, D. J. (1998). Enrichment of hen eggs with n‐3 long‐chain fatty acids and evaluation of enriched eggs in humans. American Journal of Clinical Nutrition, 68, 538–544. 9734728 10.1093/ajcn/68.3.538

[fsn31522-bib-0022] Ferrier, L. K. , Caston, L. , Leeson, S. , Squires, E. J. , Celi, B. , Thomas, L. , & Holub, B. J. (1992). Changes in serum lipids and platelet fatty acid composition following consumption of eggs enriched in alpha‐linolenic acid (LnA). Food Research International, 25, 263–268.

[fsn31522-bib-0023] Folch, J. , Lees, M. , & Sloane‐Stanley, G. H. (1957). A simple method for the isolation and purification of total lipids from animal tissues. Journal of Biological Chemistry, 226, 497–509. 13428781

[fsn31522-bib-0024] Fraeye, I. , Bruneel, C. , Lemahieu, C. , Buyse, J. , Muylaert, K. , & Foubert, I. (2012). Dietary enrichment of eggs with omega‐3 fatty acids: A review. Food Research International, 48(2), 961–969.

[fsn31522-bib-0025] Friedewald, W. T. , Levy, R. I. , & Fredrickson, D. S. (1972). Estimation of the concentration of low‐density lipoprotein cholesterol in plasma, without use of the preparative ultracentrifuge. Clinical Chemistry, 18, 499–502. 4337382

[fsn31522-bib-0026] Fuller, N. R. , Sainsbury, A. , Caterson, I. D. , & Markovic, T. P. (2015). Egg consumption and human cardio‐metabolic health in people with and without diabetes. Nutrients, 7, 7399–7420. 26404366 10.3390/nu7095344 PMC4586539

[fsn31522-bib-0027] Hargis, P. S. , & Van Elswyk, M. E. (1993). Manipulating the fatty acid composition of poultry meat and eggs for the health conscious consumer. World's Poultry Science Journal, 49, 251–264.

[fsn31522-bib-0028] Hu, F. B. , Stampfer, M. J. , Rimm, E. B. , Manson, J. E. , Ascherio, A. , Colditz, G. A. , & Hennekens, C. H. (1999). A prospective study of egg consumption and risk of cardiovascular disease in men and women. JAMA, 281(15), 1387–1394. 10217054 10.1001/jama.281.15.1387

[fsn31522-bib-0029] Jacobson, T. A. , Ito, M. K. , Maki, K. C. , Orringer, C. E. , Bays, H. E. , Jones, P. H. , … Brown, W. V. (2014). National Lipid Association recommendations for patient‐centered management of dyslipidemia: Part 1–executive summary. Journal of Clinical Lipidology, 8(5), 473–488. 25234560 10.1016/j.jacl.2014.07.007

[fsn31522-bib-0030] Jiang, Y. , Noh, S. K. , & Koo, S. I. (2001). Egg phosphatidylcholine decreases the lymphatic absorption of cholesterol in rats. Journal of Nutrition, 131, 2358–2363. 11533279 10.1093/jn/131.9.2358

[fsn31522-bib-0031] Keys, A. , Fidanza, F. , Karvonen, M. J. , Kimura, N. , & Taylor, H. L. (1972). Indices of relative weight and obesity. Journal of Chronic Diseases, 25, 329–343. 4650929 10.1016/0021-9681(72)90027-6

[fsn31522-bib-0032] Khan, S. A. , Hegde, M. V. , Wagh, U. V. , Khan, A. , & Jamadar, R. (2017). Value added eggs seem a good option for improving blood omega‐ 6: Omega‐3 ratio and heart health. Health Science, 6, 60–65.

[fsn31522-bib-0033] Khan, S. A. , Khan, A. , Khan, S. A. , Beg, M. A. , Ali, A. , & Damanhouri, G. (2017). Comparative study of fatty‐acid composition of table eggs from the Jeddah food market and effect of value addition in omega‐3 bio‐fortified eggs. Saudi Journal of Biological Sciences, 24, 929–935. 28490967 10.1016/j.sjbs.2015.11.001 PMC5415167

[fsn31522-bib-0034] Larsson, S. C. , Akesson, A. , & Wolk, A. (2015). Egg consumption and risk of heart failure, myocardial infarction, and stroke: Results from 2 prospective cohorts. American Journal of Clinical Nutrition, 102, 1007–1013. 26399866 10.3945/ajcn.115.119263

[fsn31522-bib-0035] Laudadio, V. , Lorusso, V. , Lastella, N. M. B. , Dhama, K. , Karthik, K. , Tiwari, R. , … Tufarelli, V. (2015). Enhancement of nutraceutical value of table eggs through poultry feeding strategies. International of Journal Pharmacology, 11(3), 201–212.

[fsn31522-bib-0036] Lee, J. Y. , Lewis, N. M. , Scheideler, S. E. , & Carr, T. P. (2003). Consumption of omega‐3 fatty acid‐enriched eggs and serum lipids in humans. Journal of Nutraceuticals, Functional & Medical Foods, 4, 3–13.

[fsn31522-bib-0037] Lemahieu, C. , Bruneel, C. , Ryckebosch, E. , Muylaert, K. , Buyse, J. , & Foubert, I. (2015). Impact of different omega‐3 polyunsaturated fatty acid (n‐3 PUFA) sources (flaxseed, Isochrysis galbana, fish oil and DHA Gold) on n‐3 LC‐PUFA enrichment (efficiency) in the egg yolk. Journal of Functional Foods, 19, 821–827.

[fsn31522-bib-0038] Lewis, N. M. , Schalch, K. , & Scheideler, S. E. (2000). Serum lipid response to n‐3 fatty acid enriched eggs in persons with hypercholesterolemia. Journal of the American Dietetic Association, 100, 365–367. 10719415 10.1016/S0002-8223(00)00111-5

[fsn31522-bib-0039] Lewis, N. M. , Seburg, S. , & Flanagan, N. (2000). Enriched eggs as a source of n‐3 polyunsaturated fatty acids for humans. Poultry Science, 79, 971–974. 10.1093/ps/79.7.971 10901195

[fsn31522-bib-0040] Maki, K. C. , Van Elswyk, M. E. , McCarthy, D. , Seeley, M. A. , Veith, P. E. , & Hess, S. P. , Davidson, M. H. (2003). Lipid responses in mildly hypertriglyceridemic men and women to consumption of docosahexaenoic acid‐enriched eggs. International Journal for Vitamin and Nutrition Research, 73(5), 357–368. 14639800 10.1024/0300-9831.73.5.357

[fsn31522-bib-0041] Mutungi, G. , Ratliff, J. , Puglisi, M. , Torres‐Gonzalez, M. , Vaishnav, U. , Leite, J. O. , … Fernandez, M. L. (2008). Dietary cholesterol from eggs increases plasma HDL cholesterol in overweight men consuming a carbohydrate‐restricted diet. Journal of Nutrition, 138, 272–276. 18203890 10.1093/jn/138.2.272

[fsn31522-bib-0042] Nagele, U. , Hagele, E. O. , Sauer, G. , Wiedemann, E. , Lehmann, P. , Wahlefeld, A. W. , & Gruber, W. (1984). Reagent for the enzymatic determination of serum total triglycerides with improved lipolytic efficiency. Clinical Chemistry and Laboratory Medicine, 22, 165–174. 10.1515/cclm.1984.22.2.165 6716056

[fsn31522-bib-0043] Njike, V. , Faridi, Z. , Dutta, S. , Gonzalez‐Simon, A. L. , & Katz, D. L. (2010). Daily egg consumption in hyperlipidemic adults – Effects on endothelial function and cardiovascular risk. Nutrition Journal, 9, 1–28. 20598142 10.1186/1475-2891-9-28 PMC2904713

[fsn31522-bib-0044] Noh, S. K. , & Koo, S. I. (2003). Egg sphingomyelin lowers the lymphatic absorption of cholesterol and α‐tocopherol in rats. Journal of Nutrition, 133, 3571–3576. 14608075 10.1093/jn/133.11.3571

[fsn31522-bib-0045] Oh, S. Y. , Ryue, J. , Hsieh, C. H. , & Bell, D. E. (1991). Eggs enriched in omega‐3 fatty acids and alterations in lipid concentrations in plasma and lipoproteins and in blood pressure. American Journal of Clinical Nutrition, 54, 689–695. 1897475 10.1093/ajcn/54.4.689

[fsn31522-bib-0046] Papanikolaou, Y. , Brooks, J. , Reider, C. , & Fulgoni, V. L. (2014). US adults are not meeting recommended levels for fish and omega‐3 fatty acid intake: Results of an analysis using observational data from NHANES 2003–2008. Nutrition Journal, 13, 31–44. 24694001 10.1186/1475-2891-13-31 PMC3992162

[fsn31522-bib-0047] Pourafshar, S. , Johnson, S. A. , Navaei, N. , Akhavan, N. S. , Elam, M. L. , Foley, E. , … Arjmandi, B. H. (2016). Egg consumption may be associated with improved lipid profiles and blood glucose levels in men and women with metabolic syndrome. The FASEB J, 30, 904–928.

[fsn31522-bib-0049] Rouhani, M. H. , Rashidi‐Pourfard, N. , Salehi‐Abargouei, A. , Karimi, M. , & Haghighatdoost, F. (2018). Effects of egg consumption on blood lipids: A systematic review and meta‐analysis of randomized clinical trials. Journal of the American College of Nutrition, 37(2), 99–110. 29111915 10.1080/07315724.2017.1366878

[fsn31522-bib-0050] Samimi, M. , Jamilian, M. , Asemi, Z. , & Esmaillzadeh, A. (2015). Effects of omega‐3 fatty acid supplementation on insulin metabolism and lipid profiles in gestational diabetes: Randomized, double‐blind, placebo‐controlled trial. Clinical Nutrition, 34, 388–393. 24973862 10.1016/j.clnu.2014.06.005

[fsn31522-bib-0051] Severins, N. , Mensink, R. P. , & Plat, J. (2015). Effects of lutein‐enriched egg yolk in buttermilk or skimmed milk on serum lipids & lipoproteins of mildly hypercholesterolemic subjects. Nutrition, Metabolism and Cardiovascular Diseases, 25(2), 210–217. 10.1016/j.numecd.2014.10.003 25456153

[fsn31522-bib-0052] Skórkowska‐Telichowska, K. , Kosińska, J. , Chwojnicka, M. , Tuchendler, D. , Tabin, M. , Tuchendler, R. , … Szuba, A. (2016). Positive effects of egg‐derived phospholipids in patients with metabolic syndrome. Advances in Medical Sciences, 61, 169–174. 26829066 10.1016/j.advms.2015.12.003

[fsn31522-bib-0053] Stone, N. J. , Robinson, J. G. , Lichtenstein, A. H. , Bairey Merz, C. N. , Blum, C. B. , Eckel, R. H. , … Wilson, P. W. F. (2014). 2013 ACC/AHA guideline on the treatment of blood cholesterol to reduce atherosclerotic cardiovascular risk in adults: A report of the. American College of Cardiology/American Heart Association Task Force on Practice Guidelines. Journal of the American College of Cardiology, 63(25), 2889–2934. 24239923 10.1016/j.jacc.2013.11.002

[fsn31522-bib-0054] Stupin, A. , Rasic, L. , Matic, A. , Stupin, M. , Kralik, Z. , Kralik, G. , … Drenjancevic, I. (2018). Omega‐3 polyunsaturated fatty acids‐enriched hen eggs consumption enhances microvascular reactivity in young healthy individuals. Applied Physiology, Nutrition, and Metabolism, 43(10), 988–995. 10.1139/apnm-2017-0735 29633621

[fsn31522-bib-0055] Techakriengkrai, T. , Klangjareonchai, T. , Pakpeankitwattana, V. , Sritara, P. , & Roongpisuthipong, C. (2012). The effect of ingestion of egg and low density lipoprotein (LDL) oxidation on serum lipid profiles in hypercholesterolemic women. Journal of Science Technology, 34, 173–178.

[fsn31522-bib-0056] Tousoulis, D. , Plastiras, A. , Siasos, G. , Oikonomou, E. , Verveniotis, A. , Kokkou, E. , … Stefanadis, C. (2014). Omega‐3 PUFAs improved endothelial function and arterial stiffness with a parallel antiinflammatory effect in adults with metabolic syndrome. Atherosclerosis, 232, 10–16. 24401211 10.1016/j.atherosclerosis.2013.10.014

[fsn31522-bib-0057] Warnick, G. R. , Nguyen, T. , & Albers, A. (1985). Comparison of improved precipitation methods for quantification of high‐density lipoprotein cholesterol. Clinical Chemistry, 31, 217–222. 2578337

[fsn31522-bib-0058] Weggemans, R. M. , Zock, P. L. , & Katan, M. B. (2001). Dietary cholesterol from eggs increases the ratio of total cholesterol to high‐density lipoprotein cholesterol in humans: A meta‐analysis. American Journal of Clinical Nutrition, 73(5), 885–891. 11333841 10.1093/ajcn/73.5.885

[fsn31522-bib-0059] Yashodhara, B. , Umakanth, S. , Pappachan, J. , Bhat, S. , Kamath, R. , & Choo, B. (2009). Omega‐3 fatty acids: A comprehensive review of their role in health and disease. Postgraduate Medicine, 85, 84–90. 10.1136/pgmj.2008.073338 19329703

